# An efficient cell free enzyme-based total synthesis of a meningococcal vaccine candidate

**DOI:** 10.1038/npjvaccines.2016.17

**Published:** 2016-11-15

**Authors:** Timm Fiebig, Maria Rosaria Romano, Davide Oldrini, Roberto Adamo, Marta Tontini, Barbara Brogioni, Laura Santini, Monika Berger, Paolo Costantino, Francesco Berti, Rita Gerardy-Schahn

**Affiliations:** 1 Institute for Cellular Chemistry, Hannover Medical School, Hannover, Germany; 2 GSK Vaccines, Research, Siena, Italy

## Abstract

Invasive meningococcal disease (IMD) is a global health problem and vaccination has proven the most effective way of disease control. *Neisseria meningitidis* serogroup X (*Nm*X) is an emerging threat in the African sub-Saharan meningitis belt, but no vaccine is available today. Leading vaccines against *Nm* are glycoconjugates, in which capsular polysaccharides isolated from large-scale pathogen cultures are conjugated to adjuvant proteins. Though safe and efficacious even in infants, high costs and biohazard associated with the production limit abundant application of glycoconjugate vaccines particularly in the most afflicted nations. An existing *Nm*X vaccine candidate (CPSXn-CRM_197_) produced by established protocols from *Nm*X capsule polysaccharide (CPSX) has been shown to elicit high bactericidal immunoglobulin G titres in mice. Here we describe the scalable *in vitro* synthesis of CPSXiv from chemically pure precursors by the use of recombinant *Nm*X capsule polymerase. Application of the described coupling chemistry gives CPSXiv-CRM_197_, which in mouse vaccination experiments behaves identical to the benchmark CPSXn-CRM_197_. Excluding any biohazards, this novel process represents a paradigm shift in vaccine production and a premise towards vaccine manufacturing in emerging economies.

## Introduction

*Neisseria meningitidis (Nm)* is a leading cause of bacterial meningitis and sepsis worldwide. The strictly human pathogen causes recurrent devastating epidemics in developing countries, in particular the African sub-Saharan meningitis belt.^1^ In Western World countries, *Nm* infections occur sporadically, but outbreaks have been reported caused by hyper-invasive strains in situations of crowding^2,3^ and in communities with increased risk.^4,5^ As *Nm* is the only bacterial pathogen that can spread in epidemic waves, outbreaks present a significant danger for individuals with high susceptibility, that is, small children, adolescents and elderly people.^6^

A chief problem with invasive meningococcal disease (IMD), often characterised by meningitis and sepsis, is rapid progression. IMD can lead to death within hours,^7^ leaving open an extremely short window for medical intervention. Accordingly, case-fatality rates are high, exceeding 20% even in developed countries.^8^ Moreover, survivors often suffer from fatal sequelae like limb loss, deafness and neurologic disabilities.^9^ The benefit of vaccination is obvious on this background and motivated industrial companies (GSK Vaccines, Pfizer, Baxter, Sanofi-Pasteur, Serum Institute of India^10^) and international nonprofit organisations like PATH and WHO^11,12^ to invest into the development of vaccines to combat IMD and thus improve health and life conditions in afflicted regions.

Major virulence factors of *Nm* are the capsular polysaccharides (CPS).^13^ Twelve chemically different CPSs have been identified and determine the twelve *Nm* serogroups, of which six (*Nm*A, -B, -C, -W, -Y and -X) account for virtually all cases of IMD.^13^ With the exception of *Nm*B, where the CPS (CPSB) consists of α2,8-linked polysialic acid identical to host-expressed polysialic acid,^14,15^ the CPSs coupled to adjuvant proteins provide the basis for modern vaccine formulations, the so called glycoconjugate vaccines.^16^ Currently, mono- and multivalent glycoconjugate vaccines are available for *Nm*A, -C, -W and -Y and their application has provided most promising results.^16^

Though safe and effective, glycoconjugate vaccines are under debate because of high production costs that may limit broad application in afflicted regions.^17^ Up until today, the production starts with mass fermentation of pathogens, a biohazardous step that requests the high-tech infrastructure of modern industrial plants^10^ and limits the build-up of the production infrastructure in indigent regions like the countries of the African sub-Saharan meningitis belt. However, the exigence of decentralised vaccine production has gained conspicuous actuality in the 2015 meningitis season, when an *Nm*C outbreak in Niger could not be stopped because of a shortage in vaccine provision.^18^

Considering that no licenced *Nm*X vaccine exists today and capitalising on our recent success with exploiting recombinant capsule polymerases (CPs) for *in vitro* production of bioidentical CPSs,^19,20^ we focused this study on the production of a synthetic *Nm*X vaccine. With recombinant CsxA, the CP of *Nm*X, we established reaction conditions that yielded the nature identical *in vitro* produced CPSX (henceforth referred to as CPSXiv to differentiate it from CPSXn, the polymer isolated from natural source) in homogenous quality. In a mouse vaccination experiment, a glycoconjugate vaccine prepared from CPSXiv was found to induce bactericidal antibodies at titres not different form the benchmark.^21^ The process line exemplified for *Nm*X can be easily adjusted for the production of vaccines against other *Nm* serogroups and thus should have model character for all glycoconjugate vaccines that start with the biohazardous step of pathogen fermentation.

## Results

### *In vitro* synthesis of oligosaccharide primers

**Figure 1:**
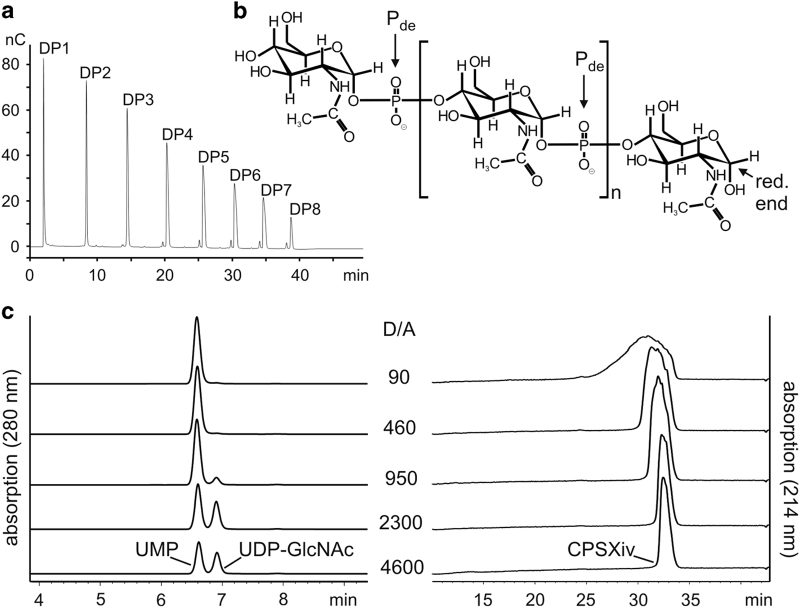
The priming oligosaccharide fraction and evaluation of reaction conditions. (**a**) HPAEC-PAD profiling shows that the primCPSXiv pool used for upscaling the CPSXiv synthesis contains the (oligo)saccharide species DP1–DP8. The minor peaks preceeding DP3–DP8 indicate the presence of small amounts of oligomers with non-reducing end phosphate groups. (**b**) Chemical structure of CPSX. P_de_, phosphodiester; red. end, reducing end. (**c**) To determine the D/A ratio that allows complete consumption of UDP-GlcNAc, CsxA reactions were carried out with a constant donor (10 mM UDP-GlcNAc) and varying acceptor (primCPSXiv) concentrations to give D/A ratios as indicated. After overnight incubation, the product spectra were analysed by HPLC-AEC. Although a small peak indicating residual UDP-GlcNAc was visible at the D/A ratio 950, conversion to UMP was complete at D/A ratio 460. CPSX, *Nm*X capsule polysaccharide; CPSXiv, *in vitro* produced CPSX; HPAEC-PAD, high-performance anion-exchange chromatography with pulsed amperometric detection; HPLC-AEC, high-performance liquid chromatography based anion exchange chromatography.

Recently we described the small scale production of CPSXiv by use of recombinant CsxA.^19^ Besides the chemically pure donor sugar UDP-*N*-acetylglucosamine (UDP-GlcNAc), short priming oligosaccharides (primCPSX) were needed to maximise the reaction efficacy and were obtained through hydrolysis of CPSXn.^19^ As it was our aim to avoid the use of *Neisseria*-derived materials to the greatest possible extent in the current upscaled reactions, we first produced primCPSXiv (Figure 1a). Still starting with primCPSXn, three consecutive CsxA catalysed reactions (reactions 1–3) were carried out. Priming oligosaccharides in reactions 2 and 3 were derived from purified and hydrolysed CPSXiv produced in reactions 1 and 2, respectively. In reactions 1–3, priming oligosaccharides were mixed with a 1,000-fold molar excess of UDP-GlcNAc, resulting in a >10^9^ fold dilution of the initially used primCPSXn. Because acidic hydrolysis cleaves the phosphodiester bond in CPSX proximal to the anomeric C-atom (Figure 1b), oligosaccharides obtained from reaction 3 were enzymatically treated to release the phosphate group from the non-reducing end and thus maximise the concentration of functional primers. Anion-exchange chromatography (AEC) used to purify this final primCPSXiv pool as well as high-performance anion-exchange chromatography with pulsed amperometric detection (HPAEC-PAD; Figure 1a) identified fragments ranging in size between DP1–DP8 (DP, degree of polymerisation), for which an average DP (avDP) of 4.5 was calculated based on ^1^H NMR analysis.^21^

### Determination of efficient reaction conditions

Because the donor-substrate UDP-GlcNAc (henceforth referred to as donor, D) is the single reaction component of appreciable economic value, completeness of conversion into CPSXiv is a critical aspect. With the primCPSXiv fraction of avDP4.5 (henceforth referred to as acceptor, A) at hand, a titration experiment was carried out to determine the D/A ratio that allowed complete consumption of UDP-GlcNAc. The donor concentration was kept at 10 mM and the acceptor increased to give D/A ratios between 4,600 and 90 (Figure 1c). The reactions were run overnight and products separated and visualised using our recently developed high-performance liquid chromatography based anion exchange chromatography (HPLC-AEC) assay.^20^ Complete conversion of UDP-GlcNAc to UMP was seen at a D/A ratio of 460. However, because the basis of the product peak, an indicator of product dispersity, had considerably broadened from D/A ratio of 950 to D/A ratio of 460, we decided for the D/A ratio of 800 to set-up medium-scale CPSXiv productions.

### Scaling up CPSXiv production and product characterisation

**Figure 2:**
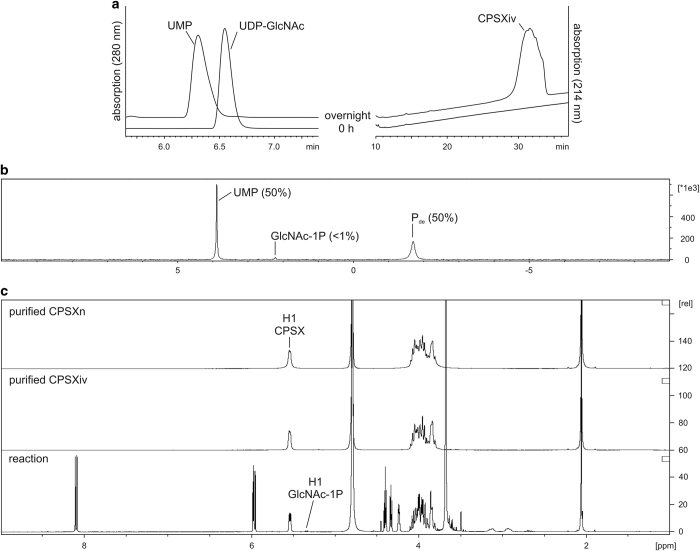
Synthesis and physico-chemical characterisation of CPSXiv. (**a**) Completeness of the reaction scaled to give 35 mg CPSXiv was monitored by HPLC-AEC. After overnight incubation, the donor (UDP-GlcNAc) peak was replaced by a UMP peak and the corresponding CPSXiv peak was recorded at 214 nm. (**b**) ^31^P NMR analysis of the reaction mixture identified two major peaks representing UMP and P_de_. Peak area integration gave equal numbers, thus confirming the quantitative integration of the donor into CPSXiv. The signal for the side product GlcNAc-1P did not exceed trace concentration. (**c**) ^1^H NMR used to control purification results and chemical identity between CPSXiv and CPSXn confirmed both, homogeneity and bio-identity of the CPSXiv fraction. CPSX, *Nm*X capsule polysaccharide; CPSXiv, *in vitro* produced CPSX; CPSXn, CPSX obtained from natural source; HPLC-AEC, high-performance liquid chromatography based anion exchange chromatography; p.p.m., parts per million.

The first upscaled reaction was calculated to give 35 mg CPSXiv. As before, UDP-GlcNAc consumption and product synthesis were visualised by HPLC-AEC (shown for start and end point in Figure 2a). After overnight incubation all UDP-GlcNAc was converted to UMP with the corresponding CPSXiv peak detected at 214 nm. A close to 100% reaction yield was confirmed when the mixture was analysed by ^31^P NMR (Figure 2b). Two main peaks representing UMP and P_de_ (phosphodiester bonds) appeared and integration of peak areas resulted in a 1:1 ratio. With only trace amounts of GlcNAc-1P, a product of a hydrolytic side activity of CsxA,^19^ the highly sensitive ^31^P NMR analysis corroborated the chosen reaction conditions. To control reproducibility, a second reaction was calculated to give 112 mg and was carried out in triplicate. The analytical HPLC-AEC profiles obtained for these reactions clearly confirmed the reliability of the CPSXiv production procedure (Supplementary Figure 1).

Because the CPSXiv production was started with ultrapure reagents and yielded close to 100% donor consumption, the product purification steps were limited to salt removal by dialysis and AEC to separate CPSXiv from UMP and GlcNAc-1P traces (data not shown). Product purity and identity were confirmed by ^1^H NMR (Figure 2c). If compared with the crude reaction mixture (bottom panel), it is immediately obvious that the two-step purification procedure delivered homogenously pure CPSXiv (middle panel). Moreover, co-analysis of CPSXn (top panel) demonstrated chemical identity of the two polymers. As a final step, we compared the size of CPSXiv and CPSXn chains using HPLC size exclusion chromatography. With a relative molecular mass of 308 kDa, CPSXiv perfectly mimicked the natural product CPSXn with a relative molecular mass of 388 kDa (Supplementary Figure 2a).

### Preparation of a CPSXiv-based glycoconjugate vaccine

**Figure 3:**
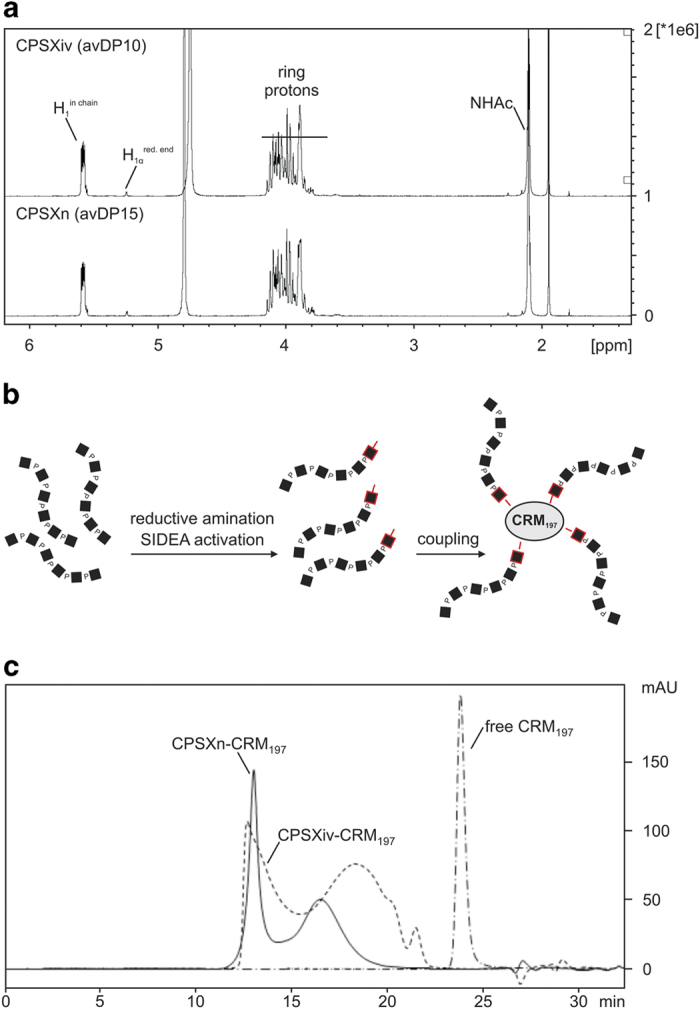
Generation of a CPSXiv-based glycoconjugate. (**a**) To ensure matching of the oligosaccharide fraction generated from CPSXiv and CPSXn, ^1^H NMR spectra were recorded and showed perfect correspondence. (**b**) Schematic representation of CPSX hydrolysis and coupling of the resulting oligosaccharides to the protein carrier CRM_197_. The reducing end sugars are marked in red. (**c**) The efficacy of oligosaccharide coupling to CRM_197_ was controlled by HPLC size exclusion chromatography with detection of the conjugates via absorption at 214 nm. Similar to the benchmark (CPSXn-CRM_197_), CPSXiv-CRM_197_ elutes in two major peaks. In line with the lower avDP of CPSXiv oligosaccharides (avDP10, Pool 2), the second peak showed a mild shift to lower molecular masses. Importantly, no free CRM_197_ was detected. CPSX, *Nm*X capsule polysaccharide; CPSXiv, *in vitro* produced CPSX; CPSXn, CPSX obtained from natural source; HPLC, high-performance liquid chromatography; p.p.m., parts per million.

For the preparation of a CPSXiv-based vaccine we precisely followed the protocol previously established for CPSXn.^21^ First, 120 mg of the synthetic polymer were hydrolysed to shorten the CPS chains and the reaction was monitored by ^31^P NMR.^22^ Hydrolysis was stopped after 270 min when ^31^P NMR analysis roughly indicated an avDP12 (Supplementary Figure 2b). The mixture was loaded onto an AEC column to remove oligosaccharides shorter than five to six repeating units. Two pools containing different oligosaccharide polydispersions were collected (Supplementary Figure 2c). Pool 2 contained 84.7 mg of oligosaccharides (70% recovery), which, as identified in an analytical HPAEC-PAD run, exhibited an overlapping dispersity with the benchmark CPSXn of avDP15 (Supplementary Figure 2d). However, with avDP10 (calculated based on ^31^P NMR analysis; see Supplementary Figure 1d), pool 2 slightly deviated from the benchmark (avDP15). Finally, the perfect congruence of the ^1^H NMR spectra obtained for the oligosaccharide fractions prepared from CPSXiv and CPSXn confirmed their chemical identity (Figure 3a).

Conjugation of CPSXiv fragments to the protein carrier CRM_197_, a non-toxic variant of the diphtheria toxin,^23^ followed the coupling technology successfully used by Micoli *et al.*^21^ to produce the CPSXn-based vaccine MenX-ADH-SIDEA-CRM_197_ (referred to as CPSXn-CRM_197_).^21^ As schematically illustrated in (Figure 3b), coupling to CRM_197_ proceeds via the reducing end of the oligosaccharides (marked by red squares). Successful coupling was confirmed by HPLC size exclusion chromatography. Similar to our benchmark, CPSXn-CRM_197_, the new glycoconjugate CPSXiv-CRM_197_ showed a bipartite elution profile in the absence of detectable amounts of free CRM_197_ (Figure 3c). However, in line with the smaller size of the oligomer fraction, the second peak was shifted right. CPSXiv-CRM_197_ was then purified by hydrophobic interaction chromatography and, as a final step in the quality control process, established protocols^21,24^ were used to determine oligosaccharide loading of CRM_197_ and control for the presence of free saccharide. These control experiments were done in parallel with CPSXn-CRM_197_ (Table 1).

### Immunogenicity testing in mice

**Figure 4:**
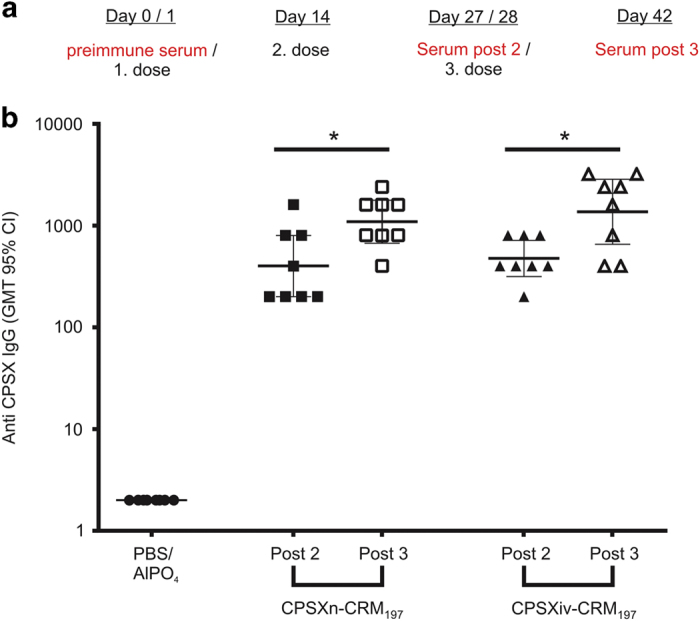
Immunogenicity of CPSXiv-CRM_197_ in comparison with CPSXn-CRM_197_. (**a**) Vaccination schedule showing the immunisation and bleeding of mice. (**b**) The anti-CPSX IgG ELISA used to determine immunisation results demonstrated comparable IgG responses to CPSX with both, the synthetic (CPSXiv-CRM_197_) and the benchmark (CPSXn-CRM_197_) vaccine. Each symbol represents an individual animal. The horizontal bars indicate the geometric mean titre and 95% confidence intervals. *Significant differences between the indicated groups with *P*<0.05 (CPSXn-CRM^197^: *P*=0.028, CPSXiv-CRM^197^: *P*=0.0284; Mann-Whitney test). CPSX, *Nm*X capsule polysaccharide; CPSXiv, *in vitro* produced CPSX; CPSXn, CPSX obtained from natural source; IgG, immunoglobulin G.

The ability of CPSXiv-CRM_197_ to elicit protective antibodies was tested in a mouse model in comparison with CPSXn-CRM_197_.^21^ Figure 4a illustrates the immunisation scheme. After the collection of pre-immune sera at day 0, female BALB/c mice (eight animals per group) were subcutaneously injected with the first dose (day 1; 1 μg saccharide content per dose, formulated in phosphate-buffered saline buffer, pH 7.2, with aluminum phosphate as adjuvant). The injections were repeated twice at day 14 and day 28. Post immunisation, sera (post) were taken at day 27 (Post 2) and day 42 (Post 3). Control animals were treated in the same scheme with phosphate-buffered saline plus adjuvant. Immunoglobulin G (IgG) titres induced were detected using our recently developed ELISA (enzyme-linked immunosorbent assay).^21^ Comparable IgG titres obtained with the two glycoconjugate vaccines indicate identical immunological properties for CPSXiv-CRM_197_ and CPSXn-CRM_197_ (Figure 4b). Moreover, it is of note that considerable IgG titres had established already after two injections (Post 2 sera) and only increased by factor 2–3 after the third vaccine application (CPSXn-CRM_197_: *P*=0.028, CPSXiv-CRM_197_: *P*=0.0284; Mann–Whitney test).

Eventually we measured bactericidal activity against *Nm*X strain Z9615. Here, sera isolated from each group and time point were pooled and rabbit serum was used as complement source.^21^ Comparable bactericidal activity (one dilution step is within the variation of the assay) measured in sera collected from CPSXiv-CRM_197_ and CPSXn-CRM_197_ immunised mice convincingly evidenced the value of the described alternative and biohazard-free protocol for the production of an *Nm*X vaccine (Table 2).

## Discussion

For the first time, we describe in this study the use of *in vitro* synthesised nature identical CPSXiv for the production of a synthetic *Nm*X glycoconjugate vaccine candidate (CPSXiv-CRM_197_). In a mouse immunisation study, CPSXiv-CRM_197_ competed with the benchmark vaccine CPSXn-CRM_197_ (ref. 21) in terms of immunogenicity and elicitation of bactericidal antibodies. We chose *Nm*X to pioneer the enzyme-catalysed vaccine production process, because this serogroup has gained prevalence worldwide^17,25–31^ and outbreaks in Niger,^25^ Uganda and Kenya,^31^ and in Togo and Burkina Faso^17,30^ have forcefully evidenced the need for a specific vaccine. Moreover, in the absence of a licensed *Nm*X vaccine, the implementation of an alternative production scheme should be expedited.

As highlighted by the Global Alliance for Vaccines and Immunisation, a key determinant towards improved health and economic conditions in low-income countries is their access to vaccines (vaccines against poverty).^32–34^ The production process described in this study should be a quantum leap in this regard. Starting from well-defined and pyrogen-free chemicals (UDP-GlcNAc and primCPSXiv) and completely excluding biohazards, the process provides a vantage ground for the build-up of the production infrastructure in indigent regions and thus increase their flexibility to reply to unforeseen needs.^18^ In addition, stockpiling of vaccines and of vaccine components (for example, oligosaccharide fractions ready for coupling) as requested by the International Coordinating Group on Vaccine Provision for Epidemic Meningitis would be facilitated with the new technology. Of utmost importance in this context is that analytical steps to guarantee safety, homogeneity and lot consistency are facilitated in the fermentation-free procedure.^35^

The described conditions allowed close to 100% conversion of the donor sugar into polymer. Small oligosaccharides that accrue in hydrolysis and sizing steps provide the optimal starters for new production rounds and a simple HPLC-based analysis enabled on-line process control. Last, but certainly not the least, the recombinant CsxA can be produced in high quality. Without any further optimisation, the enzyme purified from only 1 litre of non-pathogenic *E. coli* M15(pREP4) culture (OD_600_=3.0; ref. 19) would have sufficed to generate 8 g CPSXiv. This value is remarkable, given the fact that yields of homogenously pure CPSXn (isolated from fermented bacteria) range between 10 and 600 mg/l.^21,36–39^ Nevertheless, significant room remains for further process optimisation, in particular at the level of the recombinant enzyme. First, solid phase coupling and reuse of the enzyme in repeated production rounds would simplify and enhance the process. Second, the capsule polymerase *per se* can be engineered towards enhanced yield, solubility and/or stability,^20,40^ to acquire process-adjusted elongation modes,^41^ and, eventually, increased flexibility towards chemically functionalised acceptors. The latter could help reducing the coupling chemistry.^20^

A difference detected between the tested vaccines was a variation in the oligosaccharide dispersion (avDP10 in CPSXiv-CRM_197_ versus avDP15 in CPSXn-CRM_197_), a feature most likely attributable to the smaller scale of the CPSXiv hydrolysis reaction. Although more studies are needed to finally decide if this difference is of relevance, the fact that immunisation results did not reflect any associated variance suggests that it is negligible.

In contrast to CPSX, which represents a simple homopolymer of GlcNAc-1P repeating units, CPSs from other *Nm* serogroups are more complex and *in vitro* production would need more than one enzymatic activity. We could recently demonstrate on an analytical scale that the herein-presented technology can potentially be expanded to more complex biosynthesis systems. The acetylated CPS of *Nm*A could be produced in a one-pot reaction using three different recombinant enzymes,^20^ we have done pioneering steps towards the fermentation-free production of the dimeric CPSs of *Nm*W and Y^42,43^ and, as an example for the most complex CPS expressed by *Nm*, we also described the synthesis of the trimeric CPS of *Nm*L.^44^

Taken together, this paradigmatic evaluation of a biohazard-free protocol for the production of an efficient *Nm*X vaccine should pave the way for future decentralised glycoconjugate vaccine production and thus back up the mission for vaccines against poverty.^32^

## Materials and Methods

### Synthesis and purification of primCPSXiv

CPSXiv was synthesised *in vitro* using the recombinant capsule polymerase CsxA expressed as MBP-CsxA-His_6_ fusion construct.^19^ Hydrolysis of 0.5 mg CPSXiv (2.5 mg/ml) was performed in 50 mM sodium acetate buffer at pH 4.0 and 80 °C for 6 h. NaOH was added to readjust pH 7 before the mixture was dialysed against water (ZelluTrans, Roth, 1 kDa MWCO) and freeze-dried. Alternatively, primCPSXiv were recycled from pool 1 containing oligosaccharides <DP10 (see Supplementary Figure 1c). For the dephosphorylation of oligosaccharides, we used either acid phosphatase (Worthington Biochemical Corporation, Lakewood, NJ, USA) or calf intestinal alkaline phosphatase (CIP, NEB) and followed the manufacturer’s guidelines. Although removal of acid phosphatase from primCPSXiv was achieved with Amicon centrifugal devices (10 MWCO), CIP was removed by AEC. Individual oligosaccharides were separated on an ÄKTA-FPLC (GE Healthcare, Little Chalfont, Buckinghamshire, UK) equipped with a MonoQ HR 5/5 column (GE Healthcare) at a flow-rate of 1 ml/min. H_2_O and 1 M NaCl were used as mobile phases M_1_ and M_2_, respectively. The samples were separated using a combination of linear gradients (0 to 5% M_2_ over 1 ml, 5 to 20% M_2_ over 10 ml, 20 to 30% M_2_ over 20 ml). Saccharide-containing fractions were pooled and desalted by dialysis (ZelluTrans, Roth, 1 kDa MWCO) against a 1,000-fold excess of water. Fractions containing DP3, DP4 and DP5 were isolated and the DP was confirmed by ^1^H NMR and HPAEC-PAD as described in refs 21,22.

### Upscaling of the *in vitro* CPSX synthesis

Test reactions in 25 μl of total reaction volume were performed as described before^19^ in the presence of varying amounts of primCPSXiv to determine conditions suitable for the production of long CPSX chains and complete consumption of UDP-GlcNAc. The analysis of the reaction products by HPLC-AEC was performed following established protocols.^19,20,44^ For upscaling, 80 mg of UDP-GlcNAc were incubated with 0.6 or 2.5 nmol (62.5 or 250 μg, for overnight or 4 h reactions, respectively) MBP-CsxA-His_6_ at 37 °C in the presence of primCPSXiv in a total volume of 12.4 ml allowing the synthesis of a maximum of 35 mg CPSXiv. The synthesis of 112 mg CPSX was upscaled accordingly. UMP and other reaction constituents were removed by Tangential Flow Filtration using Vivaflow 50 membranes with 30 kDa MWCO. Alternatively, the reaction volume was reduced to 5 ml by freeze-drying and applied to an ÄKTA-FPLC (GE Healthcare) equipped with a MonoQ 10/100 GL column (GE Healthcare) at a flow-rate of 5 ml/min. H_2_O and 1 M NaCl were used as mobile phases M_1_ and M_2_, respectively. The separation was performed using a combination of linear gradients from 0 to 15% M_2_ over 24 ml and from 15 to 100% M_2_ over 224 ml. CPSX-containing fractions were pooled, dialysed against H_2_O and freeze-dried. Acidic hydrolysis was performed for 4.5 h as described above and the avDP was estimated by ^31^P NMR.^22^ The hydrolysate was loaded onto a Q Sepharose column (GE Healthcare) equilibrated with binding buffer (5 mM sodium acetate, pH 7.0). Small oligosaccharides (<DP5-6) were removed with binding buffer containing 200 mM NaCl (pool I; Supplementary Figure 1c) and longer oligosaccharides were eluted with binding buffer containing 1 M NaCl.

### Generation and immunogenicity of CPSXn-CRM_197_

#### Immunisation protocol, serum analysis, ELISA and rSBA

All methods concerning the generation and characterisation of CPSXiv-CRM_197_ as well as the immunisation protocol and serum analysis were performed as described before.^21^ *Nm*X strain Z9615 was kindly provided by Gerd Pluschke, Swiss Tropical and Public Health Institute, Molecular Immunology, Basel, Switzerland. The animal studies were performed in accordance with the Italian law, approved by the local Animal Ethics Committee, and authorised by the Italian Ministry of Health.

## Figures and Tables

**Table 1:** Comparative characterisation of CPSXiv-CRM_197_ and CPSXn-CRM_197_

	*Saccharide/protein ratio (w/w)*	*Saccharide/protein ratio (mol/mol)*	*Unconjugated saccharide %*
CPSXiv-CRM_197_	0.2	4.1	<6.0
CPSXn-CRM_197_	0.3	4.1	<3.5

Abbreviations: CPSX, *Nm*X capsule polysaccharide; CPSXiv, *in vitro* produced CPSX; CPSXn, CPSX obtained from natural source.

The saccharide/protein ratios (mol/mol) for CPSXiv-CRM_197_ and CPSXn-CRM_197_ were calculated using the average molecular weight of the respective fraction (avDP10 for CPSXiv-CRM_197_ and avDP15 for CPSXn-CRM_197_).

**Table 2:** Rabbit serum bactericidal activity assay

*Group*	*Post 2 (pooled sera)*	*Post 3 (pooled sera)*
PBS/AlPO_4_	<4	<4
CPSXn-CRM_197_	2,048	8,192
CPSXiv-CRM_197_	1,024	4,096

Abbreviations: CPSX, *Nm*X capsule polysaccharide; CPSXiv, *in vitro* produced CPSX; CPSXn, CPSX obtained from natural source; PBS, phosphate-buffered saline.

Post 2 and Post 3 sera were pooled for the respective groups of mice. Bactericidal titres are defined as the reciprocal serum dilution that gives a 50% decrease of colony-forming units after 60 min incubation in the reaction mixture, compared with the mean number of colony-forming units in the control reactions at time point zero.

## References

[bib1] Stephens, D. Biology and pathogenesis of the evolutionarily successful, obligate human bacterium *Neisseria meningitidis*. Vaccine 27(Suppl 2): B71–B77 (2009). 19477055 10.1016/j.vaccine.2009.04.070 PMC2712446

[bib2] Broderick, M. P., Phillips, C. & Faix, D. Meningococcal disease in US military personnel before and after adoption of conjugate vaccine. Emerg. Infect. Dis. 21, 377–379 (2015). 25625525 10.3201/eid2102.141037 PMC4313647

[bib3] Hill, D. J., Griffiths, N. J., Borodina, E. & Virji, M. Cellular and molecular biology of *Neisseria meningitidis* colonization and invasive disease. Clin. Sci. (Lond) 118, 547–564 (2010). 20132098 10.1042/CS20090513 PMC2830671

[bib4] Kratz, M. M. et al. Community-Based Outbreak of *Neisseria meningitidis* Serogroup C Infection in Men who Have Sex with Men, New York City, New York, USA, 2010-2013. Emerg. Infect. Dis. 21, 1379–1386 (2015). 26197087 10.3201/eid2108.141837 PMC4517726

[bib5] Kupferschmidt, K. Infectious diseases. Bacterial meningitis finds new niche in gay communities. Science 341, 328 (2013). 23888010 10.1126/science.341.6144.328

[bib6] Caesar, N. M., Myers, K. A. & Fan, X. *Neisseria meningitidis* serogroup B vaccine development. Microb. Pathog. 57, 33–40 (2013). 23416222 10.1016/j.micpath.2013.02.003

[bib7] Cartwright, K. A. & Ala’Aldeen, D. A. *Neisseria meningitidis*: clinical aspects. J. Infect. 34, 15–19 (1997). 9120319 10.1016/s0163-4453(97)80004-7

[bib8] Tan, L. K., Carlone, G. M. & Borrow, R. Advances in the development of vaccines against *Neisseria meningitidis*. N. Engl. J. Med. 362, 1511–1520 (2010). 20410516 10.1056/NEJMra0906357

[bib9] Nadel, S. Prospects for eradication of meningococcal disease. Arch. Dis. Child 97, 993–998 (2012). 22984187 10.1136/archdischild-2012-302036 PMC3512348

[bib10] Costantino, P., Rappuoli, R. & Berti, F. The design of semi-synthetic and synthetic glycoconjugate vaccines. Expert Opin. Drug Discov 6, 1045–1066 (2011). 22646863 10.1517/17460441.2011.609554

[bib11] Tiffay, K., Jodar, L., Kieny, M. P., Socquet, M. & Laforce, F. M. The Evolution of the Meningitis Vaccine Project. Clin. Infect. Dis. 61(Suppl 5): S396–S403 (2015). 26553666 10.1093/cid/civ594 PMC4639496

[bib12] Laforce, F. M., Konde, K., Viviani, S. & Preziosi, M. P. The Meningitis Vaccine Project. Vaccine 25(Suppl 1): A97–A100 (2007). 17521780 10.1016/j.vaccine.2007.04.049

[bib13] Tzeng, Y. L., Thomas, J. & Stephens, D. S. Regulation of capsule in *Neisseria meningitidis*. Crit Rev. Microbiol. 42, 759–772 (2015). 26089023 10.3109/1040841X.2015.1022507 PMC4893341

[bib14] Lo, H., Tang, C. M. & Exley, R. M. Mechanisms of avoidance of host immunity by *Neisseria meningitidis* and its effect on vaccine development. Lancet Infect. Dis. 9, 418–427 (2009). 19555901 10.1016/S1473-3099(09)70132-X

[bib15] Eckhardt, M. et al. Molecular characterization of eukaryotic polysialyltransferase-1. Nature 373, 715–718 (1995). 7854457 10.1038/373715a0

[bib16] Pace, D. Glycoconjugate vaccines. Expert Opin. Biol. Ther. 13, 11–33 (2013). 22992106 10.1517/14712598.2012.725718

[bib17] Xie, O., Pollard, A. J., Mueller, J. E. & Norheim, G. Emergence of serogroup X meningococcal disease in Africa: need for a vaccine. Vaccine 31, 2852–2861 (2013). 23623866 10.1016/j.vaccine.2013.04.036

[bib18] Maurice, J. Vaccine shortage threatens spread of meningitis in Niger. Lancet 385, 2241 (2015). 26088485 10.1016/S0140-6736(15)61050-9

[bib19] Fiebig, T. et al. Functional expression of the capsule polymerase of *Neisseria meningitidis* serogroup X: a new perspective for vaccine development. Glycobiology 24, 150–158 (2014). 24259400 10.1093/glycob/cwt102

[bib20] Fiebig, T. et al. Molecular cloning and functional characterization of components of the capsule biosynthesis complex of *Neisseria meningitidis* serogroup A: toward *in vitro* vaccine production. J. Biol. Chem. 289, 19395–19407 (2014). 24849599 10.1074/jbc.M114.575142 PMC4094051

[bib21] Micoli, F. et al. Development of a glycoconjugate vaccine to prevent meningitis in Africa caused by meningococcal serogroup X. Proc. Natl Acad. Sci. USA 110, 19077–19082 (2013). 24191022 10.1073/pnas.1314476110 PMC3839747

[bib22] Berti, F. et al. Relative stability of meningococcal serogroup A and X polysaccharides. Vaccine 30, 6409–6415 (2012). 22921741 10.1016/j.vaccine.2012.08.021

[bib23] Bröker, M., Costantino, P., DeTora, L., McIntosh, E. D. & Rappuoli, R. Biochemical and biological characteristics of cross-reacting material 197 CRM197, a non-toxic mutant of diphtheria toxin: use as a conjugation protein in vaccines and other potential clinical applications. Biologicals 39, 195–204 (2011). 21715186 10.1016/j.biologicals.2011.05.004

[bib24] Micoli, F. et al. Meningococcal X polysaccharide quantification by high-performance anion-exchange chromatography using synthetic N-acetylglucosamine-4-phosphate as standard. Anal. Biochem. 442, 259–261 (2013). 23938776 10.1016/j.ab.2013.08.001

[bib25] Boisier, P. et al. Meningococcal meningitis: unprecedented incidence of serogroup X-related cases in 2006 in Niger. Clin. Infect. Dis. 44, 657–663 (2007). 17278055 10.1086/511646

[bib26] Chen, C. et al. A first meningococcal meningitis case caused by serogroup X *Neisseria meningitidis* strains in China. Chin. Med. J. (Engl. ) 121, 664–666 (2008). 18466690

[bib27] Fazio, C. et al. *Neisseria meningitidis* serogroup X sequence type 2888, Italy. Emerg. Infect. Dis. 16, 359–360 (2010). 20113588 10.3201/eid1602.091553 PMC2958031

[bib28] Vicente, D., Esnal, O. & Perez-Trallero, E. Fatal *Neisseria meningitidis* serogroup X sepsis in immunocompromised patients in Spain. Virulence of clinical isolates. J. Infect. 64, 184–187 (2012). 22108049 10.1016/j.jinf.2011.11.009

[bib29] Kilic, A. et al. *Neisseria meningitidis* serogroup X sequence type 767 in Turkey. J. Clin. Microbiol. 48, 4340–4341 (2010). 20739496 10.1128/JCM.01417-10 PMC3020828

[bib30] Delrieu, I. et al. Emergence of epidemic *Neisseria meningitidis* serogroup X meningitis in Togo and Burkina Faso. PLoS One 6, e19513 (2011). 21625480 10.1371/journal.pone.0019513 PMC3098835

[bib31] Mutonga, D. M. et al. Epidemiology and risk factors for serogroup X meningococcal meningitis during an outbreak in western Kenya, 2005-2006. Am. J. Trop. Med. Hyg. 80, 619–624 (2009). 19346388

[bib32] MacLennan, C. A. & Saul, A. Vaccines against poverty. Proc. Natl Acad. Sci. USA 111, 12307–12312 (2014). 25136089 10.1073/pnas.1400473111 PMC4151718

[bib33] Shen, A. K. et al. Country ownership and Gavi transition: comprehensive approaches to supporting new vaccine introduction. Health Aff. (Millwood) 35, 272–276 (2016). 26858380 10.1377/hlthaff.2015.1418

[bib34] Berkley, S. Make vaccine coverage a key UN health indicator. Nature 526, 165 (2015). 26450023 10.1038/526165a

[bib35] Jones, C. Glycoconjugate vaccines: the regulatory framework. Methods Mol. Biol. 1331, 229–251 (2015). 26169744 10.1007/978-1-4939-2874-3_14

[bib36] Chilukuri, S. R. et al. Process development and immunogenicity studies on a serogroup ‘X’ Meningococcal polysaccharide conjugate vaccine. Biologicals 42, 160–168 (2014). 24411634 10.1016/j.biologicals.2013.12.001

[bib37] Robinson, J. A. & Apicella, M. A. Isolation and characterization of *Neisseria meningitidis* Groups A, C, X and Y polysaccharide antigens. Infect. Immun. 1, 8–14 (1970). 16557699 10.1128/iai.1.1.8-14.1970 PMC415847

[bib38] Bundle, D. R., Jennings, H. J. & Kenny, C. P. Studies on the group-specific polysaccharide of *Neisseria meningitidis* serogroup X and an improved procedure for its isolation. J. Biol. Chem. 249, 4797–4801 (1974). 4211095

[bib39] Xie, O. et al. Characterization of size, structure and purity of serogroup X *Neisseria meningitidis* polysaccharide, and development of an assay for quantification of human antibodies. Vaccine 30, 5812–5823 (2012). 22835740 10.1016/j.vaccine.2012.07.032

[bib40] Keys, T. G., Berger, M. & Gerardy-Schahn, R. A high-throughput screen for polysialyltransferase activity. Anal. Biochem. 427, 60–68 (2012). 22579847 10.1016/j.ab.2012.04.033

[bib41] Keys, T. G. et al. Engineering the product profile of a polysialyltransferase. Nat. Chem. Biol. 10, 437–442 (2014). 24727899 10.1038/nchembio.1501

[bib42] Romanow, A. et al. Biochemical and biophysical characterization of the sialyl-/hexosyltransferase synthesizing the meningococcal serogroup W135 heteropolysaccharide capsule. J. Biol. Chem. 288, 11718–11730 (2013). 23439648 10.1074/jbc.M113.452276 PMC3636861

[bib43] Romanow, A. et al. Dissection of hexosyl- and sialyltransferase domains in the bifunctional capsule polymerases from *Neisseria meningitidis* W and Y defines a new sialyltransferase family. J. Biol. Chem. 289, 33945–33957 (2014). 25342753 10.1074/jbc.M114.597773 PMC4256332

[bib44] Litschko, C. et al. The capsule polymerase CslB of *Neisseria meningitidis* serogroup L catalyzes the synthesis of a complex trimeric repeating unit comprising glycosidic and phosphodiester linkages. J. Biol. Chem. 290, 24355–24366 (2015). 26286750 10.1074/jbc.M115.678094 PMC4591819

