# Supplementary material for: An efficient cell free enzyme-based total synthesis of a meningococcal vaccine candidate
Source: NPJ Vaccines. 2016 Nov 15;1:16017–. doi: 10.1038/npjvaccines.2016.17 (PMC5707881; doi:10.1038/npjvaccines.2016.17)
Supplement: Supplementary Information [file npjvaccines201617-s1.doc]

**An efficient cell free enzyme-based total synthesis of a meningococcal vaccine candidate**

Timm Fiebig^a*^, Maria Rosaria Romano^b*^, Davide Oldrini^b^, Roberto Adamo^b^, Marta Tontini^b^, Barbara Brogioni^b^, Laura Santini^b^, Monika Berger^a^, Paolo Costantino^b^, Francesco Berti^b§^ and Rita Gerardy-Schahn^a§^

*^a^Institute for Cellular Chemistry, Hannover Medical School, Carl-Neuberg-Strasse 1, 30625 Hannover, Germany*

*^b^GSK Vaccines, Research, Via Fiorentina 1, 53100 Siena, Italy*

*^*^Authors contributed equally to this study*

^§^Corresponding authors: Prof. Dr. Rita Gerardy-Schahn, Hannover Medical School, Carl-Neuberg Str. 1, 30625 Hannover, Germany; Phone: +49 511 532 9802; Fax: +49 511 532 8801; E-mail: gerardy-schahn.rita@mh-hannover.de.

Dr. Francesco Berti, GSK Vaccines, Research, Via Fiorentina 1, 53100 Siena, Italy; Phone: +39 0577 243895; Fax: +39 0577 243564; E-mail: francesco.x.berti@gsk.com.

**Running title:** An *in vitro* synthesized vaccine against *Nm*X

The study was financed by LOM (impact oriented funding) funds to the Institute for Cellular Chemistry.

**Supplementary Figures**

**
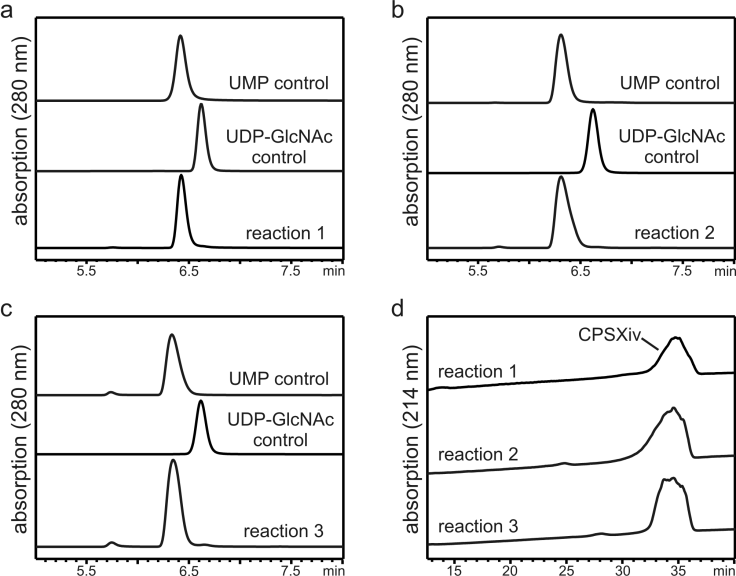
**

**Supplementary Figure 1. HPLC-AEC analysis of a medium scale CsxA-catalysed reaction.** In order to control reproducibility of medium scale CsxA reactions, the reaction calculated to produce 112 mg CPSXiv was set-up in triplicate. (**a-c**) UV (280 nm) spectra recorded at the reaction endpoint demonstrate complete consumption of UDP-GlcNAc in each case. Only the second reaction product UMP was detected. UMP and UDP-GlcNAc were used as reference to control elution conditions. (**d**) In each reaction, the chromatogram recorded at 214 nm showed the expected CPSXiv-peak.

**
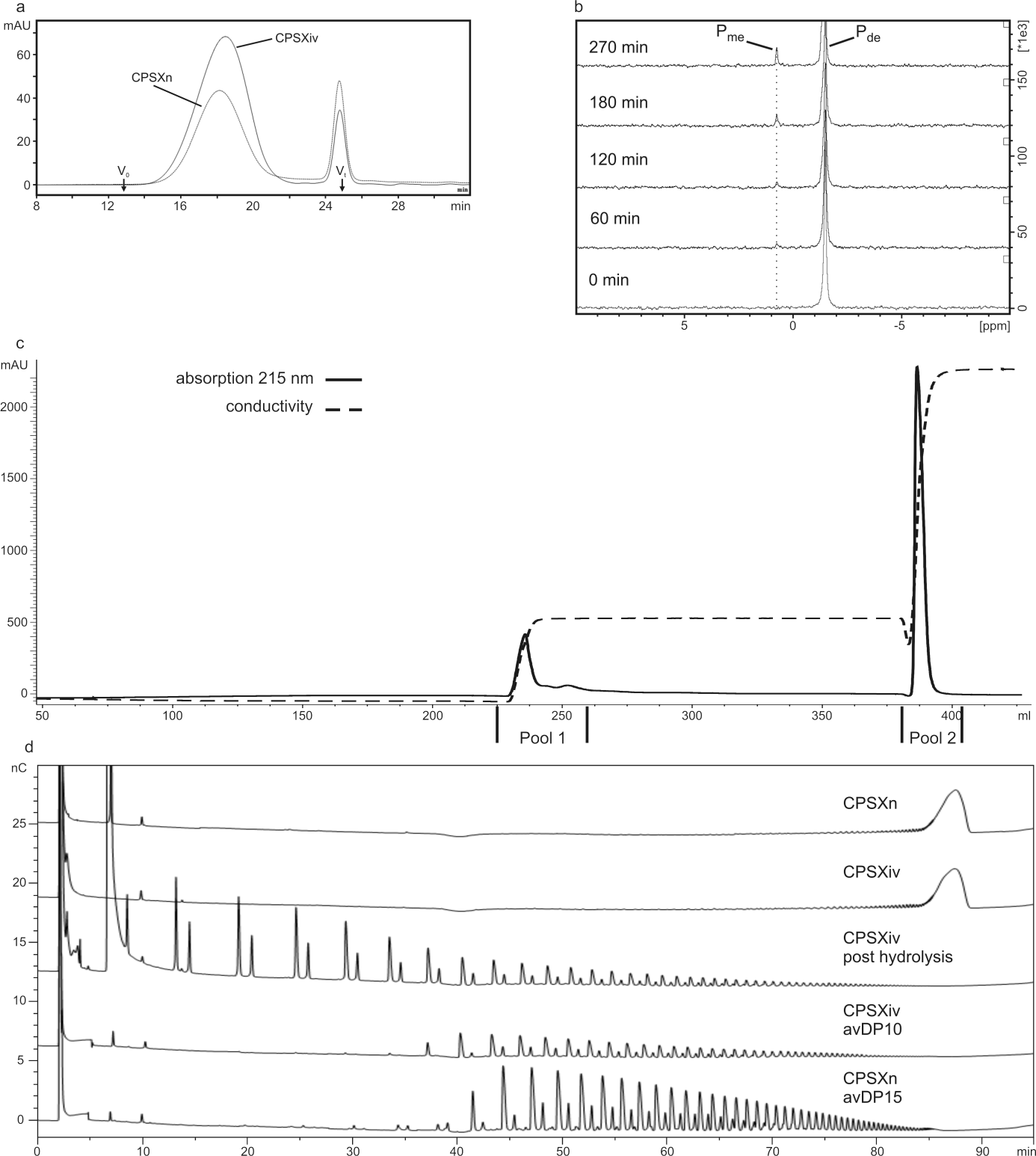
**

**Supplementary Figure 2. Physico-chemical analysis of CPSXiv in comparison to CPSXn.** (**a**) The relative molecular weight of CPSXiv and CPSXn was determined using HPLC-SEC and sample detection at 214 nm. V_0_ and V_t_ of the column were determined using dextran and sodium azide, respectively. (**b**) During hydrolysis of CPSXiv, ^31^P NMR spectra were recorded at the indicated time points and signals of internal phosphodiester groups (P_de_) and phosphomonoester end-groups (P_me_) were integrated and used for the calculation of avDPs (avDP = [(P_de_/P_me_) + 1]). (**c**) AEC was used to fractionate the hydrolysed CPSXiv. Pool 1 contained oligosaccharides smaller than DP5-6 and Pool 2 contained the oligosaccharides that were subsequently coupled to CRM_197_. (**d**) HPAEC-PAD profiling of CPSXiv poly- and oligosaccharides at various stages of the process in comparison to samples obtained by applying identical procedures onto CPSXn. The dispersity of long CPSXiv is indiscernible from long CPSXn (upper chromatograms). After hydrolysis oligosaccharides obtained from CPSXiv were, based on ^31^P NMR data, calculated to attain avDP10, while oligosaccharides obtained with CPSXn attained avDP15. The dispersity of both oligosaccharide samples (lower chromatograms) was however in good correspondence.
